# Multiple-marker phylogeny and morphological evidence reveal two new species in Steccherinaceae (Polyporales, Basidiomycota) from Asia

**DOI:** 10.3897/mycokeys.78.57823

**Published:** 2021-04-05

**Authors:** Ting Cao, Jia-Rui Yu, Trang Thị Thu Nguyễn, Hai-Sheng Yuan

**Affiliations:** 1 CAS Key Laboratory of Forest Ecology and Management, Institute of Applied Ecology, Chinese Academy of Sciences, Shenyang 110164, China; 2 University of the Chinese Academy of Sciences, Beijing 100049, China; 3 Department of Microbiology, Faculty of Biology and Biotechnology, University of Science, Vietnam; National University, Ho Chi Minh City, Vietnam

**Keywords:** Hydnaceous fungus, molecular phylogeny, polypores, taxonomy, wood-inhabiting fungi

## Abstract

Two new wood-inhabiting fungi, *Mycorrhaphium subadustum***sp. nov.** and *Trullella conifericola***sp. nov.**, are proposed and described from Asia based on ITS, nrLSU and *tef1* molecular phylogeny and morphological characteristics. *Mycorrhaphium subadustum* is characterized by a stipitate basidiocarp, velutinate pileal surface concentrically zoned, hydnoid hymenophore, a dimitic hyphal system in spine trama and monomitic in context, absence of gloeocystidia, presence of cystidioles and the non-amyloid, cylindrical to ellipsoid basidiospores. *Trullella conifericola* is characterized by a laterally stipitate basidiocarp with flabelliform to semicircular pileus, hirtellous pileal surface with appressed coarse hair and concentrically zoned and sulcate, tiny pores (10–12 per mm), a dimitic hyphal system, absence of any type of cystidia, short clavate basidia and thin-walled, smooth, cylindrical to allantoid basidiospores. Phylogenetic analyses based on a three-marker dataset were performed using maximum likelihood and Bayesian inference methods. The two new species formed isolated lineages with full support in Steccherinaceae. The distinguishing characters of the two new species as well as allied species are discussed, and a key to species of *Mycorrhaphium* is provided.

## Introduction

Steccherinaceae Parmasto was typified by the genus *Steccherinum* Gray (1968). It belongs to the residual polyporoid clade of the Polyporales Gäum. (Basidiomycota). It is a distinct and well-defined group based on phylogenetic evidence (Miettinen et al. 2012; Binder et al. 2013). Steccherinaceae includes around 23 genera according to Zmitrovich (2018). The taxa in this family show highly variable morphological and anatomical features. For instance, the basidiocarps range from resupinate (e.g. *Junghuhnia* Corda.) to pileate (e.g. *Austeria* Miettinen and *Flabellophora* G. Cunn.), and the hymenophore can be poroid (e.g. *Citripora* Miettinen) or hydnoid (e.g. *Mycorrhaphium* Maas Geest. and *Steccherinum* Gray). The hyphal system ranges from monomitic (e.g. *Caudicicola* Miettinen, M. Kulju & Kotir. and *Elaphroporia* Z.Q. Wu & C.L. Zhao), dimitic (e.g. *Antrodiella* Ryvarden & I. Johans.) to trimitic (e.g. *Metuloidea* G. Cunn.). Any type of cystidia can be absent (e.g. *Frantisekia* Spirin & Zmitr.) or take the form of gloeocystidia (e.g. *Antella* Miettinen and *Butyrea* Miettinen) or encrusted cystidia (e.g. *Flaviporus* Murrill). The basidiospores are typically cylindrical, allantoid (e.g. *Nigroporus* Murrill and *Trullella* Zmitr.) or ellipsoid (e.g. *Steccherinum* Gray). Nevertheless, the members of the family also share several characters including the white-rot nutritional mode, small pores or densely arranged spines, smooth and relatively small basidiospores, and mainly cyanophilic but inamyloid hyphae (Gray 1821; Corda 1842; Murrill 1905; Maas Geesteranus 1962; Cunningham 1965; Ryvarden and Johansen 1980; Spirin et al. 2007; Yuan and Dai 2009; Yuan and Wu 2012; Yuan et al. 2012; Yuan 2014; Miettinen and Ryvarden 2016; Kotiranta et al. 2017; Wu et al. 2018; Zmitrovich 2018).

Morphological and phylogenetic analyses have provided more accurate identification and contributed to the definition of the taxonomic status of the genera in Steccherinaceae. In recent years, phylogenetic analysis based on multi-marker data has been widely used in the taxonomy of these fungi (He and Dai 2012; Miettinen et al. 2012; Binder et al. 2013; Dai et al. 2014; Miettinen and Ryvarden 2016; Justo et al. 2017; Kotiranta et al. 2017; Westphalen et al. 2018; Yuan et al. 2018; Yuan et al. 2020).

The species of the Steccherinaceae are widely distributed all over the world. During the investigation of specimens in Steccherinaceae from Asia, several specimens which represent two undescribed species were found. The morphological and molecular features showed that they belong to the genus *Mycorrhaphium* and *Trullella*. In this study, we describe them as two new species based on morphological characteristics and three-marker phylogenetic analyses.

## Material and methods

### Morphological studies

The studied specimens were deposited at the herbarium of the Institute of Applied Ecology, Chinese Academy of Sciences (IFP). Microscopic procedures followed Yuan and Qin (2018). Microscopic observations were made on tissue sections mounted in cotton blue and Melzer’s reagent to test for any amyloid and/or dextrinoid reactions (cotton blue: 0.1 mg Methyl blue (SIGMA, PCode: 1001545602) dissolved in 60 g pure lactic acid; Melzer’s reagent: 1.5 g KI (potassium iodide), 0.5 g I (crystalline iodine), 22 g chloral hydrate, distilled water 20 mL). The following abbreviations are used in the text: KOH = 2.5% potassium hydroxide; CB = cotton blue; CB+/– = cyanophilous/acyanophilous; IKI = Melzer’s reagent; IKI– = neither amyloid nor dextrinoid; L_m_ = mean spore length (arithmetic average of all spores); W_m_ = mean spore width (arithmetic average of all spores); Q = variation in the ratios of L_m_/W_m_ between specimens studied, and n = total number of spores measured from a given number of specimens. Sections were studied at magnifications up to ×1000 using a Nikon Eclipse E600 microscope (Tokyo, Japan) with phase-contrast illumination, and dimensions were estimated subjectively with an accuracy of 0.1 μm. Microscopic drawings were made with the aid of a drawing tube. The spores’ measurements excluded the apiculus, and 5% of the measurements at each end of the range are given in parentheses. The spores’ measurements were made with a Nikon SMZ 645 stereomicroscope. Special colour terms are from Kornerup and Wanscher (1981).

### Molecular procedures and phylogenetic analyses

DNA was extracted from dried herbarium specimens with a Thermo Scientific Phire Plant Direct PCR kit (Thermo Fisher Scientific, Waltham, Massachusetts, USA) according to the manufacturer’s instructions and was used for the polymerase chain reaction (PCR). Nuclear ribosomal RNA markers were used to determine the phylogenetic position of the new species. The internal transcribed spacer (ITS) was amplified with the primers ITS4 (5' TCCTCCGCTTATTGATATGC 3') and ITS5 (5' GGAAGTAAAAGTCGTAACAAGG 3'); LR0R (5' ACCCGCTGAACTTAAGC 3') and LR7 (5' TACTACCACCAAGATCT 3') for partial nrLSU; 983F (5' GCYCCYGGHCAYCGTGAYTTYAT 3') and 2218R (5' ATGACACCRACRGCRACRGTYTG 3') for *tef1* (White et al.1990; Gardes and Bruns 1993; Rehner and Buckley 2005; Matheny et al. 2007).

PCR reactions were performed in 30 μL reaction mixtures containing 15 μL of 2×Phire Plant PCR buffer, 0.6 μL Phire Hot Start II DNA Polymerase, 1.5 μL of each PCR primer (10 μM), 10.5 μL double deionized H_2_O (ddH_2_O), and 0.9 μL template DNA. The PCR thermal cycling program condition was set as follows: initial denaturation at 95 °C for 5 min, followed by 34 cycles at 95 °C for 30 s, the annealing temperatures were as follows: 58.9 °C for ITS4/ITS5, 47.2 °C for LR0R/LR7, 57.6 °C for 983F/2218R, then 72 °C for 20 s, and a final extension at 72 °C for 7 min. PCR amplification was confirmed on 1% agarose electrophoresis gel stained with ethidium bromide (Stöger et al. 2006) and sequenced at the Beijing Genomics Institute (BGI) with the same primers as used in PCR. The newly generated DNA sequences were assembled and manually modified with the software DNAMAN8 (Lynnon Biosoft, Quebec, Canada). The sequences quality control followed the guidelines by Nilsson et al. (2012). All newly obtained sequences were submitted to GenBank (Sayers et al. 2020). Sequences from allied genera were based on the studies of Miettinen et al. (2012), Yuan (2014) and Westphalen et al. (2019) or found in GenBank (http://www.ncbi.nlm.gov) using the BLAST option and downloaded (Table 1). DNA alignments were performed using the MAFFT v.7.471 online service (https://mafft.cbrc.jp/alignment/server/index.html; Katoh et al. 2019). Intron regions of *tef1* as well as low-homology regions of ITS1 and ITS2 were removed before phylogenetic analyses, and the sequence datasets were combined using BioEdit v 7.2.6 (Hall 2005).

**Table 1.** Specimens and sequences used in this study. Type specimens are indicated as superscript T and the newly generated sequences in this study are in bold.

Species	GenBank No.			Specimen/culture voucher	Locality	References
	ITS	nrLSU	*tef1*			
*Antella americana* (Ryvarden & Gilb.) Ryvarden	JN710509	JN710509	JN710711	KHL 11949	Sweden	Miettinen et al. 2012
*A. americana*	EU232186	EU232270	–	HHB 4100-Sp	USA	GenBank Database
*A. chinensis* (H.S. Yuan) Miettinen	JX110844	KC485542	–	Dai 9019^T^	China	Yuan 2013
* chinensis *	JX110843	KC485541	–	Dai 8874^T^	China	Yuan 2013
*A. niemelaei* (Vampola & Vlasák) Miettinen	AF126876	–	–	Renvall 3218	Finland	Johannesson et al. 2000
*A. niemelaei*	AF126877	–	–	Haikonen 14727	Finland	Johannesson et al. 2000
*A. lactea* H.S. Yuan	KC485530	KC485548	–	Yuan 5720^T^	China	Yuan 2014
*A. lacteal*	KC485532	KC485550	–	Yuan 5757^T^	China	Yuan 2014
*A. semisupina* (Berk. & M.A. Curtis) Ryvarden	JN710521	JN710521	–	X242	Canada	Miettinen et al. 2012
*Antrodiella* sp.	JN710523	JN710523	–	Núñez 1040	Japan	Miettinen et al. 2012
*A. stipitata* H.S. Yuan & Y.C. Dai	KC485525	KC485544	–	Yuan 5640	China	Yuan 2014
*Atraporiella neotropica* Ryvarden	HQ659221	HQ659221	–	Miettinen X1021	Belize	Miettinen et al. 2012
*Austeria citrea* (Berk.) Miettinen	JN710511	JN710511	–	X1171	New Zealand	Miettinen et al. 2012
*Butyrea luteoalba* (P. Karst.) Miettinen	JN710558	JN710558	JN710719	isolate 5403	Estonia	Miettinen et al. 2012
*B. japonica* (Núñez & Ryvarden) Miettinen & Ryvarden	JN710556	JN710556	JN710718	isolate 10202^T^	Japan	Miettinen et al. 2012
*B. japonica*	KC485536	KC485553	–	Li 1648	China	Yuan 2014
*Cabalodontia queletii* (Bourdot & Galzin) Piątek	AF141626	AF141626	–	FCUG 722	Sweden	GenBank Database
*Citripora bannaensis* Miettinen	JN710526	JN710526	–	OM9999^T^	China	Miettinen et al. 2012
*Climacocystis borealis* (Fr.) Kotl. & Pouzar	JN710527	JN710527	–	KHL 13318	Estonia	Miettinen et al. 2012
*Elaphroporia ailaoshanensis* Z.Q. Wu & C.L. Zhao	MG231568	MG748854	–	CLZhao 595^T^	China	Wu et al. 2018
* ailaoshanensis *	MG231572	MG748855	–	CLZhao 596	China	Wu et al. 2018
*Etheirodon fimbriatum* (Pers.) Banker	JN710530	JN710530	–	KHL 11905	Sweden	Miettinen et al. 2012
*Flabellophora* sp1	JN710533	JN710533	–	Miettinen 14305	Indonesia	Miettinen et al. 2012
*Flabellophora* sp2	JN710534	JN710534	–	Miettinen 11443	Indonesia	Miettinen et al. 2012
*Flabellophora* sp3	JN710535	JN710535	–	Syamsi NOM677	Indonesia	Miettinen et al. 2012
*Flabellophora* sp4	JN710536	JN710536	–	Ryvarden 34508	USA	Miettinen et al. 2012
*Flabellophora* sp.	**MT269765**	**MT259330**	**MT793111**	Yuan 12794	China	This study
*F.* sp.	**MT269766**	**MT259331**	**MT793112**	Yuan 12796	China	This study
*Flaviporus brownii* (Humb.) Donk	KY175008	KY175008	KY175022	MCW 362/12	Ecuador	Westphalen et al. 2018
*F. brownie*	JN710538	JN710538	–	X462	Australia	Miettinen et al. 2012
*liebmannii* (Fr.) Ginns	JN710539	JN710539	–	X249	China	Miettinen et al. 2012
*F. liebmannii*	KC502914	–	–	Yuan 1766	China	Yuan 2014
*Frantisekia mentschulensis* (Pilát ex Pilát) Spirin	FJ496670	FJ496728	–	BRNM 710170	Czech Republic	Tomšovský et al. 2010
*F. mentschulensis*	JN710544	JN710544	–	isolate 1377	Australia	Miettinen et al. 2012
*F. ussurii* Y.C. Dai & Niemelä	KC485526	–	–	Dai 8249	China	Yuan 2014
*F. ussurii*	KC485527	KC485545	–	Wei 3081	China	Yuan 2014
*Junghuhnia crustacea* (Jungh.) Ryvarden	JN710553	JN710553	–	X626	Indonesia	Miettinen et al. 2012
*J. micropora* Spirin, Zmitr. & Malysheva	JN710559	JN710559	JN710720	Spirin 2652	Russia	Miettinen et al. 2012
*Lamelloporus americanus*	JN710567	JN710567		Læssœ 10119	Ecuador	Miettinen et al. 2012
*Loweomyces fractipes* (Berk. & M.A. Curtis) Jülich	KX378866	KX378866	–	MT 13/2012	Brazil	Westphalen et al. 2016
*L. spissus* Westph., Tomšovský & Rajchenb.	KX378869	KX378869	–	MCW 488/14	Brazil	Westphalen et al. 2016
*L. tomentosus* Westph., Tomšovský & Rajchenb.	KX378870	KX378870	–	MCW 366/12^T^	Brazil	Westphalen et al. 2016
*L. wynneae* (Berk. & Broome) Jülich	JN710604	JN710604	–	X1215	Denmark	Miettinen et al. 2012
*Metuloidea cinnamomea* (Iturr. & Ryvarden) Miettinen & Ryvarden	KU926963	–	–	X1228^T^	Venezuela	Miettinen and Ryvarden 2016
*M. fragrans* (A. David & Tortic) Miettinen	KC858281	–	–	LE295277	Russia	GenBank Database
*M. murashkinskyi* (Burt) Miettinen & Spirin	JN710588	JN710588	–	X449	Russia	Miettinen et al. 2012
*M. rhinocephala* (Berk.) Miettinen	JN710562	JN710562	–	X460	Australia	Miettinen et al. 2012
*Mycorrhaphium adustum* (Schwein.) Maas Geest.	JN710573	JN710573	JN710727	KHL12255	USA	Miettinen et al. 2012
*M. hispidum* Westph. & Miettinen	MH475306	MH475306	MH475317	MCW 363/12^T^	Brazil	Westphalen et al. 2019
*M. hispidum*	MH475307	MH475307	MH475318	MCW 429/13	Brazil	Westphalen et al. 2019
*M. subadustum*	KC485537	KC485554	–	Dai 10173^T^	China	Yuan 2014
*M. subadustum*	**MW491378**	**MW488040**	**MW495253**	Yuan 12976^T^	China	This study
*Nigroporus vinosus* (Berk.) Murrill	JX109857	JX109857	JX109914	BHS2008-100	USA	Binder et al. 2013
*N. vinosus*	JN710575	JN710575	–	X839	Indonesia	Miettinen et al. 2012
N. cf. vinosus	**MT681923**	**MT675108**	**MT793113**	Yuan 12916	China	This study
*N. stipitatus* Douanla-Meli & Ryvarden	JN710574	JN710574	–	X546^T^	Cameroon	Miettinen et al. 2012
*Skeletocutis novae-zelandiae* (G. Cunn.)P.K. Buchanan & Ryvarden	JN710582	JN710582	–	Ryvarden 38641	New Zealand	Miettinen et al. 2012
*Steccherinum aridum* Svrček	JN710583	JN710583	–	Bureid 110510	Norway	Miettinen et al. 2012
S. cf. ciliolatum	JN710585	JN710585	–	Ryvarden 47033	Estonia	Miettinen et al. 2012
*S. meridionale* (Rajchenb.) Westphalen, Tomšovský & Rajchenberg	KY174992	KY174992	KY175019	MR 284	Chile	Westphalen et al. 2018
*S. neonitidum* Westphalen & Tomšovský	KY174990	KY174990	KY175017	MCW 371/12^T^	Brazil	Westphalen et al. 2018
*S. ochraceum* (Pers. ex J.F. Gmel.) Gray	JN710590	JN710590	JN710730	KHL 11902	Brazil	Miettinen et al. 2012
*S. robustius* (J. Erikss. & S. Lundell) J. Erikss.	JN710591	JN710591	–	G1195	Sweden	Miettinen et al. 2012
*S. straminellum* (Bres.) Melo	JN710597	JN710597	–	KH Larsson 13849	France	Miettinen et al. 2012
*Trullella conifericola*	**MT269764**	–	–	Cui 2851^T^	China	This study
*T. conifericola*	**MT269760**	**MT259326**	**MT793109**	Yuan 12655^T^	Vietnam	This study
*T. conifericola*	**MT269761**	**MT259327**	**MT793110**	Yuan 12657^T^	Vietnam	This study
*T. dentipora* (Ryvarden & Iturr.) Zmitr.	JN710512	JN710512	–	X200^T^	Venezuela	Miettinen et al. 2012
*T. duracina* (Pat.) Zmitr.	MH475309	MH475309	–	MCW 410/13	Brazil	Westphalen et al. 2019
*T. duracina*	MH475310	MH475310	–	RP 96	Brazil	Westphalen et al. 2019
*T. meridae* (Miettinen & Ryvarden) Zmitr.	KY980668	KY980676	–	AS 2150	Brazil	GenBank Database
*T. meridae*	JN710513	JN710513	–	X290^T^	Venezuela	Miettinen et al. 2012
*T. polyporoides* (Ryvarden & Iturr.) Zmitr.	JN710602	JN710602	–	X510^T^	Venezuela	Miettinen et al. 2012
*Xanthoporus syringae* (Parmasto) Audet	JN710607	JN710607	–	Jeppson 2264	Sweden	Miettinen et al. 2012
*X. syringae*	AY789078	AY684166	DQ059049	AFTOL-ID 774	China	Miettinen et al. 2012

Bayesian analysis and Maximum likelihood were applied to the ITS + nrLSU + *tef1* dataset. All characters were weighted, and gaps were treated as missing data. Bayesian analysis with MrBayes 3.2.7 (Ronquist et al. 2012) implemented the Markov Chain Monte Carlo (MCMC) technique. The combined dataset was divided into seven partitions: ITS1, 5.8S, ITS2, nrLSU and *tef1* 1^st^, 2^nd^ as well as 3^rd^ codon positions. The best-fit models selected were K80+G for ITS1, GTR+I+G for 5.8S, JC+G for ITS2, GTR+I+G for nrLSU, JC for *tef1* 1^st^, TrNef+G for *tef1* 2^nd^ and GTR+I+G *tef1* 3^rd^ which were determined by the jModelTest 2.1.10 (Darriba et al. 2012) based on the Corrected Akaike Information Criterion (AICc). Four simultaneous Markov chains were run with 10 million generations and starting from random trees and keeping one tree every 100^th^ generation until the average standard deviation of split frequencies was below 0.01. The value of burn-in was set to discard 25% of trees when calculating the posterior probabilities. Bayesian posterior probabilities were obtained from the 50% majority rule consensus of the trees kept. A Maximum Likelihood (ML) analysis uses the seven-partitions’ database which is the same as Bayesian analysis and performed in RAxML v8.2.4 (Stamatakis 2014). The best tree was obtained by performing 1000 rapid bootstrap inferences followed by a thorough search for the most likely tree (Stamatakis et al. 2008). Phylogenetic trees were checked and modified in FigTree 1.4 (Rambaut 2012). The combined dataset and trees were deposited in TreeBASE (No. S27633).

## Results

### Phylogenetic analyses

**Figure 1.**
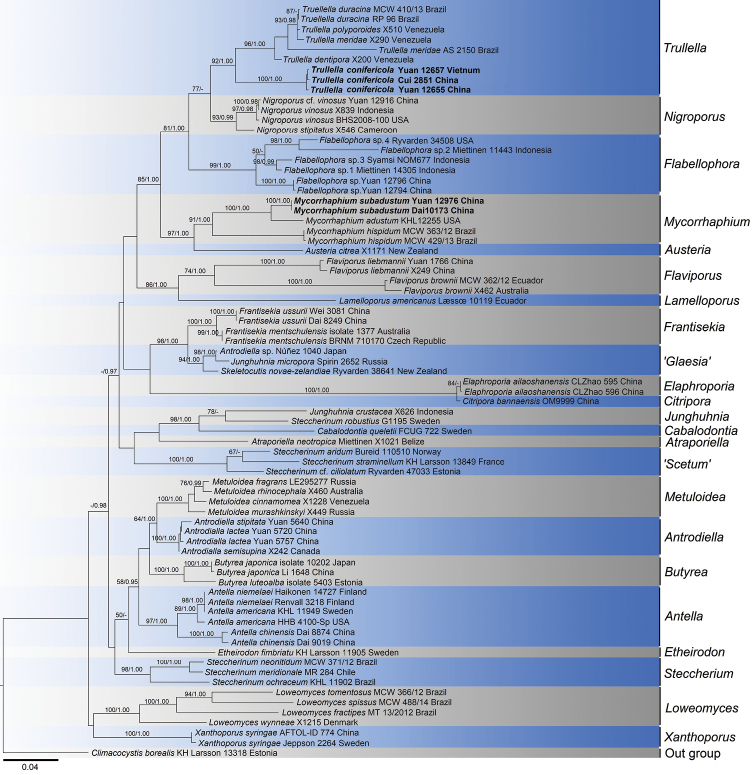
Maximum likelihood tree based on the combined ITS + nrLSU + *tef1* sequence dataset illustrating the phylogeny of *Mycorrhaphium subadustum* and *Trullella conifericola* and related taxa in Steccherinaceae. The new species are in bold. Branches are labelled with maximum likelihood bootstrap higher than 50% and Bayesian posterior probabilities more than 0.95.

Multiple-marker analyses provide an advantage of accurately and promptly discovering a new species or genus (Taylor et al. 2000). Therefore, we used three markers in our dataset which included 75 ITS, 68 nrLSU and 20 *tef1*. The combined dataset includes two species belonging to the genera *Mycorrhaphium* and *Trullella* respectively, and other 69 samples from 23 allied genera. *Climacocystis borealis* (Fr.) Kotl. & Pouzar was used as the outgroup. The data matrix comprised 163 sequences and had an aligned length of 2121 bases. Bayesian analysis resulted in an average standard deviation of split frequencies = 0.004878. The maximum likelihood and Bayesian analyses produced similar topologies and therefore, only the ML tree is shown in Figure 1.

The two new species *Mycorrhaphium subadustum* and *Trullella conifericola* were both defined with three markers and they form full-support (100% ML and 1.00 BPP) isolated lineages respectively in this study. The new species *M. subadustum* clustered together with *Mycorrhaphium* spp. and form a subclade with American *M. adustum*. In case of another new species *T. conifericola*, although the material of *T. conifericola* Cui 2851 was only provided with ITS sequences, it showed a high similarity of ITS to the other two samples (Yuan 12657 and Yuan 12655) with 99.59% and 98.77% respectively. Furthermore, the morphological and anatomical features, distribution and the coniferous-saprophytic habit suggested it represented an individual which belongs to *T. conifericola*. Three samples of *T. conifericola* get together with another six samples from the *Trullella* clade with support 92% in ML and 1.00 BPP. The phylogenetic tree obtained in this study is similar to that of Miettinen et al. (2012). All the species were divided into 23 main clades which include *Antella*, *Antrodiella*, *Atraporiella*, *Austeria*, *Butyrea*, *Cabalodontia*, *Citripora*, *Elaphroporia*, *Etheirodon*, *Flabellophora*, *Flaviporus*, *Frantisekia*, ‘*Glaesia*’, *Junghuhnia*, *Lamelloporus*, *Loweomyces*, *Metuloidae*, *Mycorrhaphium*, *Nigroporus*, ‘*Scetum*’, *Steccherinum*, *Trullella* and *Xanthoporus*. It is notable that the genera *Austeria*, *Flabellophora*, *Mycorrhaphium*, *Nigroporus* and *Trullella* formed a large clade in Steccherinaceae with a strong support (85% ML and 1.00 BPP).

### Taxonomy

#### Mycorrhaphium subadustum

Taxon classificationFungiPolyporalesMeruliaceae

T. Cao & H.S. Yuan sp. nov.

46260127-E1F9-5242-8BDA-D06A564E3E5E

838509

Figures 2 , 3

##### Diagnosis.

Basidiocarps stipitate; pileus semicircular to dimidiate; pileal surface velutinate, concentrically zonate, pileal margin yellowish white; hymenophore hydnoid. Hyphal system dimitic in spine trama and monomitic in context; generative hyphae with clamp connections; cystidia and gloeocystidia absent, cystidiols present. Basidiospores cylindrical to allantoid, CB–, IKI–.

##### Holotype.

**China.** Liaoning Province, Huanren County, Laotudingzi Nature Reserve, on fallen branch of angiosperm, 4.VIII.2018, *Yuan 12976* (**holotype**IFP 019374).

##### Etymology.

*Subadustum* (Lat.), referring to the affinity with *M. adustum*.

##### Description.

***Basidiocarps*** annual, stipitate, solitary or imbricate, corky to soft fibrous, without odor and taste when fresh, light in weight when dry. *Pilei* semicircular to dimidiate, 2.5–4.5 cm wide and 0.3 cm thick. *Pileal surface* velutinate, smooth, concentrically zonate, yellowish white to greyish orange (4A2–5B4); margin acute, yellowish white (4A2). Hymenophore hydnoid; spines crowded, evenly distributed, greyish orange (5B4), fibrous, subulate to terete, straight to somewhat flexuous, solitary or confluent, up to 1 mm long, 5–7 per mm; sterile margin smooth, yellowish grey (4B2), up to 2 mm wide. Context yellowish white (3A2), leathery, azonate, homogeneous, up to 0.5 mm thick. Stipe up to 3 cm long, 1 cm wide, straight and base inflated, surface tomentum eventually glabrous, brownish orange (5C4).

***Hyphal structure*.** Hyphal system monomitic in context, dimitic in spine trama; generative hyphae often with clamp connections and simple septate occasionally present; skeletal hyphae thick-walled to subsolid, CB+, IKI–; tissues pale yellow in KOH.

***Context*.** Generative hyphae with clamp connections, colorless, thin- to slightly thick-walled, frequently branched, 3–5 µm diam; skeletal hyphae absent.

**Figure 2.**
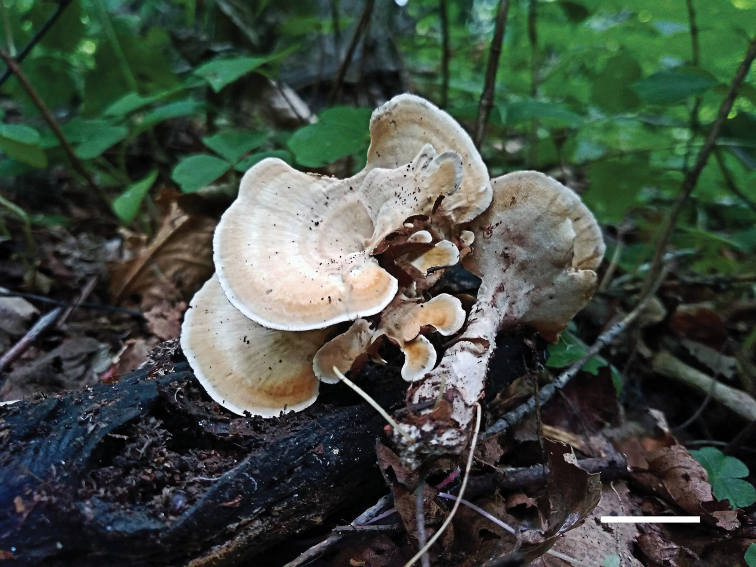
Basidiocarps of *Mycorrhaphium subadustum* (IFP 019374, holotype). Scale bar: 10 mm.

***Spines*.** Generative hyphae often with clamp connections, simple-septate occasionally present, colorless, thin- to slightly thick-walled, moderately branched, 2.5–4 µm diam; skeletal hyphae thick-walled to subsolid, unbranched, subparallel along the spine, 3–5 µm diam. Gloeocystidia absent; cystidioles present among the basidia, fusiform, 8–12 × 1.5–3 µm. Basidia clavate, with a basal clamp and four sterigmata, 8–13.5 × 2–3.5 µm; basidioles in shape similar to basidia, but slightly smaller.

***Basidiospores*** cylindrical to ellipsoid, colorless, thin-walled, smooth, CB–, IKI–, (3.5–)3.8–4.0(4.2) × (1.5–)1.8–1.9(–2.0) µm, L_m_ = 3.89 µm, W_m_ = 1.83 µm, Q = 2.13–2.17 (n = 60/2).

##### Type of rot.

White rot.

##### Distribution.

In temperate zones.

##### Additional specimen examined.

**China.** Jilin Province, Antu Country, Changbai Mountain Nature Reserve, Huangsongpu, on fallen branch of angiosperm, 2.VIII.2008, *Dai 10173* (IFP 008336).

**Figure 3.**
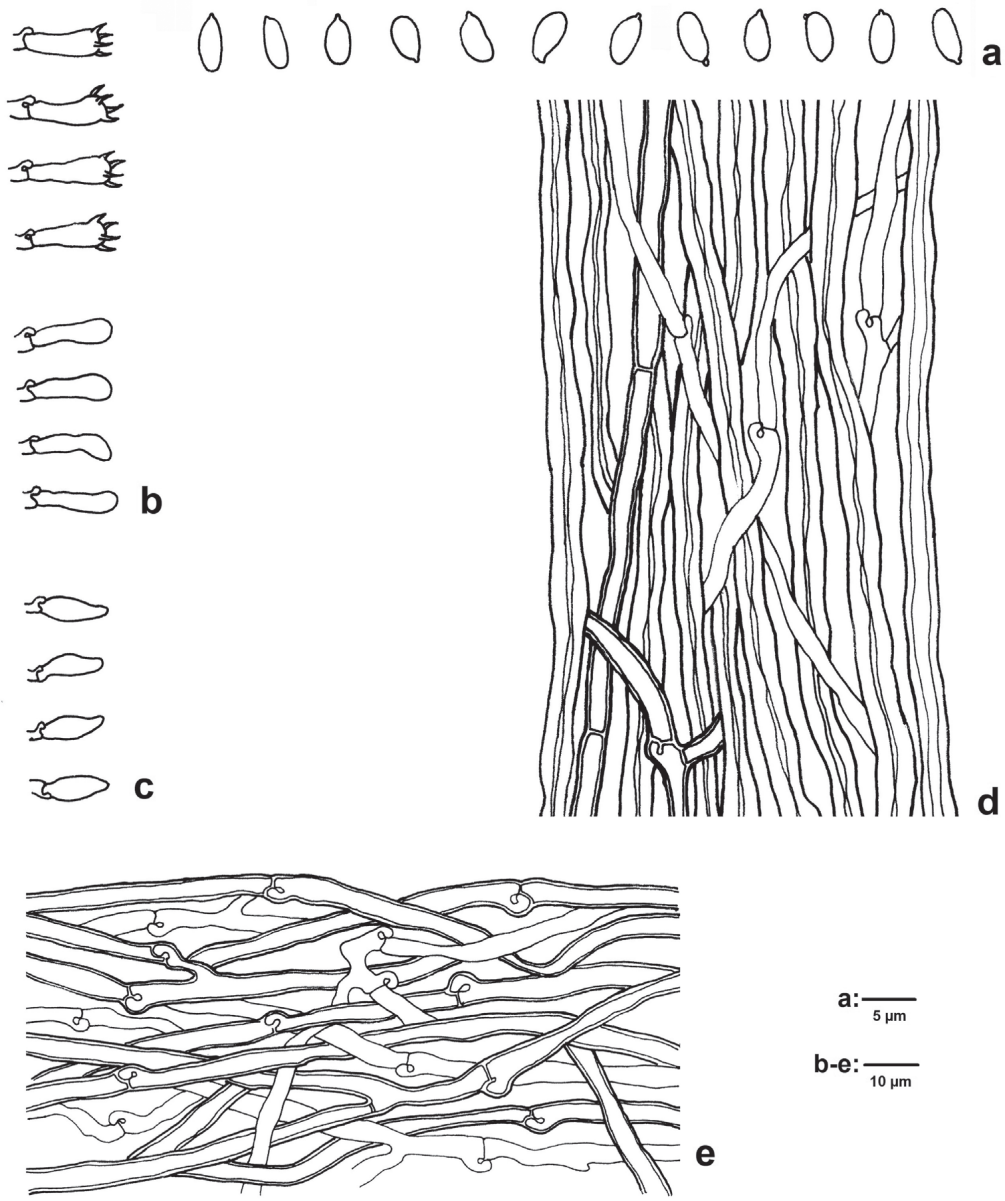
Microscopic structures of *Mycorrhaphium subadustum* (IFP 019374, holotype) **a** Basidiospores **b** Basidia and basidioles **c** cystidioles **d** Hyphae from spine trama **e** Hyphae from context.

#### Trullella conifericola

Taxon classificationFungiPolyporalesMeruliaceae

T. Cao & H.S. Yuan sp. nov.

0065542D-FD06-53CA-8221-D55B1F89FE2E

836287

Figures 4 , 5

##### Diagnosis.

Basidiocarps annual, sessile or laterally stipitate; pileus flabelliform to semi-circular; pileal surface hirtellous, with appressed coarse hair, concentrically zonate and sulcate; pores round to angular. Hyphal system dimitic; generative hyphae with clamp connections; skeletal hyphae CB+, IKI–. Basidiospores cylindrical to allantoid, thin-walled.

##### Holotype.

**Vietnam.** Lam dong Province, Lac Duong District, Lac Duong District, Bidoup Nui Ba National Park, on fallen branch of *Pinus kesiya*, 15.X.2017, *Yuan 12655* (**holotype**IFP 019372).

##### Etymology.

*Conifericola* (Lat.), referring to growth on the coniferous substrate.

##### Description.

***Basidiocarps*** annual, sessile or laterally stipitate, solitary to imbricate, without special odor or taste, leathery when fresh, shrinking, hard corky and light in weight upon drying. *Pileus* flabelliform to semi-circular, applanate, projecting 4–10 cm and 1 cm thick at the base; pileal surface hirtellous, with appressed coarse hair, concentrically zonate and sulcate, alternating white and greyish orange (6A1–6B3) when fresh, yellowish white (2A2/3A2/4A2) and nearly azonate when dry; margin acute, drying involute and wavy. *Pore surface* light orange (5A4), shiny; pores round to angular, tiny, 10–12 per mm, hardly visible to the naked eye; dissepiments entire; sterile margin ca. 1 mm wide. *Context* color paler than pores and upper surface, yellowish white (2A2–3A2), soft corky, azonate, 0.5–1.5 mm thick. *Tubes* non-stratified, concolorous with pore surface, dense, ca. 1.5 mm thick when dry. *Stipe* round, glabrous and smooth, light yellow to greyish yellow (4A4–4B5), 0.5–2 cm long and 2–4 mm in diam, dense and homogenous.

**Figure 4.**
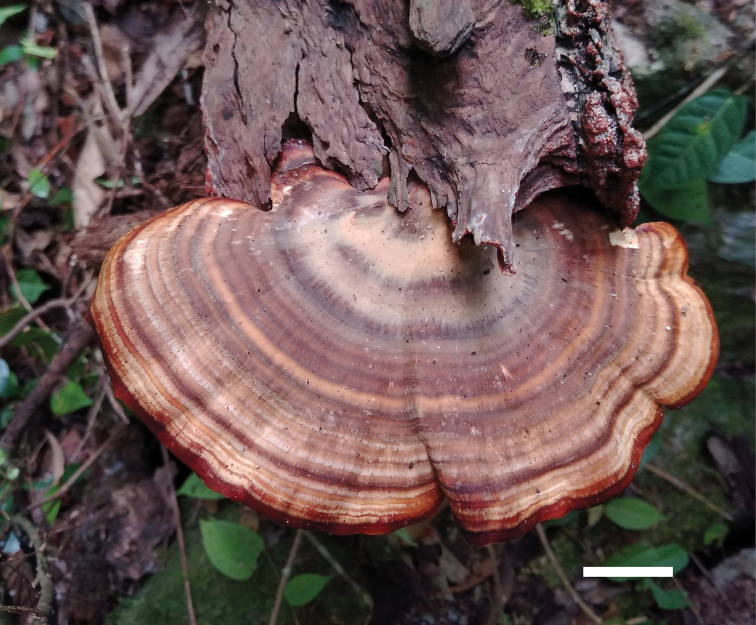
Basidiocarps of *Trullella conifericola* (IFP 019372, holotype). Scale bar: 10 mm.

***Hyphal structure*.***Hyphal system* dimitic: generative hyphae bearing clamp connections, skeletal hyphae CB+, IKI–; tissues unchanged in KOH.

***Context*.** Dominated by generative hyphae, interwoven; generative hyphae hyaline, thin- to slightly thick-walled, clamp connections abundant, frequently branched, 2.5–5.5 μm diam; skeletal hyphae hyaline, thick-walled with a wide lumen, unbranched, 1.5–5 μm diam.

***Tubes*.** Dominated by skeletal hyphae, interwoven; generative hyphae hyaline, thin- to slightly thick-walled, moderately branched, 2–4 µm diam; skeletal hyphae hyaline, thick-walled to semisolid, straight to flexuous, unbranched, 1.5–3.5 µm diam. *Cystidia* or other sterile hymenial elements absent. *Basidia* short 8–15 × 4–5.5 µm, clavate, 4-sterigmata of 0.5–1 µm in length, with a clamp connection at base; basidioles similar to basidia in shape, but slightly smaller.

***Basidiospores*.** Cylindrical to allantoid, slightly curved, hyaline, thin-walled, smooth, CB–, IKI–, (4.0–)4.1–5.5(–5.8) × (1.6–)1.8–2.3(–2.5) µm, L_m_ = 4.94 µm, W_m_ = 2.09 µm, Q = 2.36–2.45 (n = 60/2).

##### Ecology.

On fallen gymnosperm branch, causing a white rot.

##### Distribution.

In high altitude area of subtropical to tropical zones.

##### Additional specimens examined.

**China**. Fujian Prov., Wuyishan Forest Park, on fallen trunk of *Pinus kesiya*, 16.IX.2005, *Cui 2851* (IFP 000645). **Vietnam**. Lam dong Province, Lac Duong District, Bidoup Nui Ba National Park, on fallen branch of *Pinus kesiya*, 15.X.2017, *Yuan 12657* (IFP 019373).

**Figure 5.**
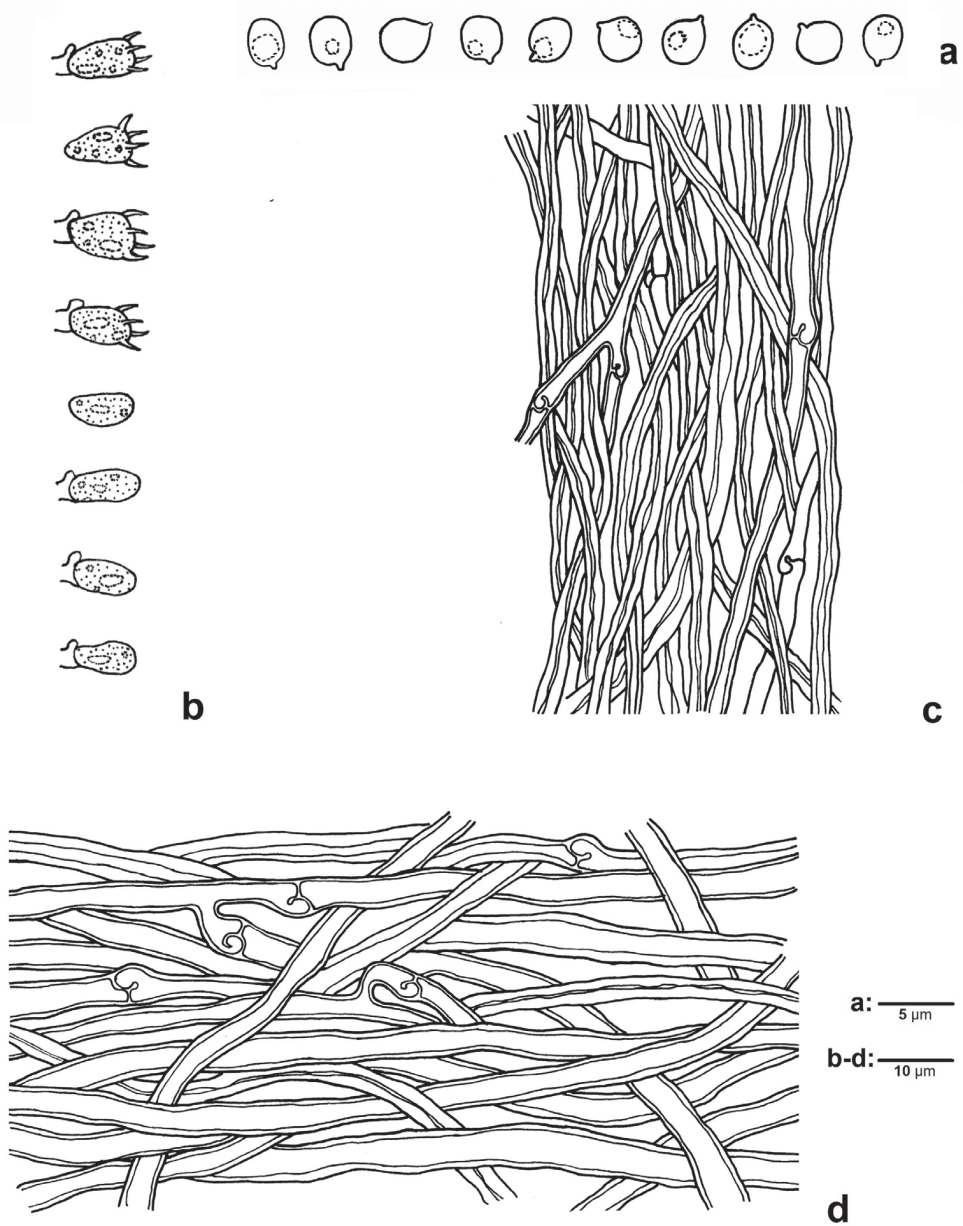
Microscopic structures of *Trullella conifericola* (IFP 019372, holotype) **a** basidiospores **b** basidia and basidioles **c** hyphae from trama **d** hyphae from context.

## Discussion

The phylogenetic profiling showed that the new species *Mycorrhaphium subadustum* as well as *Trullella conifericola* are nested in the Steccherinaceae which belongs to the residual polyporoid clade (Miettinen et al. 2012; Binder et al. 2013; Zmitrovich 2018; Westphalen et al. 2019) where they emerge robustly supported isolated lineages. Furthermore, these lineages are supported by morphological characteristics.

*Mycorrhaphium* was recommended by Maas Geesteranus (1962) and typified by *M. adustum*. The two samples of the new species *M. subadustum* (Yuan 12976 and Dai 10173) clustered in *Mycorrhaphium* clade, were both collected on fallen branches of angiosperm from northeast of China. The similarity of ITS and nrLSU sequences between the two samples of *M. subadustum* are 99.00% as well as 99.64% respectively, and they form a full-support isolated lineage which is closely related to *M. adustum*, the type species of the genus. The type material of *M. subadustum* Yuan 12976 have a 95.56% similarity of ITS sequences with the American *M. adustum* KHL12255. Morphologically, *M. subadustum* is similar to *M. adustum* in having the velutinate and concentrically zonate pileal surface, presence of clamps and simple septa, a dimitic hyphae system in spine trama and monomitic in context, absence of cystidia as well as gloeocystidia and the non-amyloid basidiospores. However, *M. adustum* often have a dark-colored pileal margin, which is distinctly different from the yellowish white ones of *M. subadustum*. Anatomically, the new species can be differentiated from *M. adustum* by the slender generative hyphae in context (3–5 µm vs. 4–6.3 µm), cyanophilous hyphae and presence of cystidiols (Maas Geesteranus 1962; Ryvarden 1989; Westphalen et al. 2019).

*Mycorrhaphium* embraced nine species (http://www.indexfungorum.org, 2020) and among which there are others two species described from Asia: *Mycorrhaphium sessile* H.S. Yuan & Y.C and *M. stereoides* Maas Geest. *M. sessile* is a species described from China, but the characteristics such as the sessile basidiocarps and presence of gloeocystidia can differentiate it from *M. subadustum* (Yuan and Dai 2009). *Mycorrhaphium stereoides* is related to *M. subadustum* in having stipitate basidiocarps, hydnoid hymenophore, a monomitic hyphal system in context and dimitic in spines, but differs from it by the presence of gloeocystidia and the larger basidiospores (4–6.3 × 2.5–3.8 µm) (Maas Geesteranus 1971). The North Europe *Mycorrhaphium pusillum* (Brot.) Maas Geest. is closely related to *M. subadustum* in having the stipitate basidiocarps as well as pale colored and zonate pileal surface, but differs it by the presence of gloeocystidia, absence of clamps and the broader basidiospores (Q = 1.52 in *M. pusillum* vs. 2.13–2.17 in *M. subadustum*) (Tervonen et al. 2015). *Mycorrhaphium ursinum* Decock & Ryvarden is a new species from African; its habit of growing on the soil can be distinguished from *M. subadustum*. Ryvarden (1989) as well as Mossebo and Ryvarden (2003) have provided keys to a part of species in *Mycorrhaphium* and after which several new taxa have been described. We provide a new key to the whole described species (except *M. ursinum*) of the genus in this study.

In the phylogenetic tree, nine samples of *Trullella* species which include the new species *T. conifericola* form the clade with strong support (92% ML and 1.00 BPP). *Trullella* is agenus which was originally proposed as ‘*Trulla*’ by Miettinen and Ryvarden (2016) and renamed by Zmitrovich (2018). *Trullella conifericola* is quite an extraordinary species in the genus because of its coniferous-saprophytic habit. The type species of *Trullella*, *T. dentipora* (Ryvarden & Iturr.) Zmitr., was described from South America. *Trullella dentipora*, together with the other species of the genus, inhabits dead angiosperm trees (e.g. *Quercus* and *Cecropia peltata*) (Patouillard 1902; Murrill 1907; Miettinen and Ryvarden 2016). Morphologically and anatomically, *T. conifericola* resembles others *Trullella* spp. in having sessile or laterally stipitate basidiocarps, mostly small and regular pores, a dimitic hyphal structure, nearly monomitic in the context, and curved cylindrical spores. However, the new species can be distinctly differentiated from others species by having a hirtellous pileal surface with appressed coarse hair, larger spores than those of previous *Trullella* species (L_m_ = 4.94 µm and W_m_ = 2.09 µm in *T. conifericola* vs L_m_ = 4.00–4.77 µm and W_m_ = 1.39–1.91 µm in others *Trullella* spp.), and inhabiting fallen gymnosperm branches. *Trullella* composed of six species as of now, and the key to these species was provided by Miettinen and Ryvarden (2016).

Besides, the genera *Mycorrhaphium* and *Trullella* together with *Austeria*, *Flabellophora* and *Nigroporus* form a large clade in the phylogenetic tree with strong support (85% ML and 1.00 BPP), and share similar morphological features, including zonate or sulcate pileal surfaces, tiny pores or dense spines and a context that is entirely or almost monomitic. They form a distinct subgroup in the Steccherinaceae.

### Key to species of worldwide *Mycorrhaphium*

**Table:** 

1	Hymenophore hydnoid	**2**
–	Hymenophore poroid	***M. hispidum* Westph. & Miettinen**
2	Spores less than 3.5 µm long	**3**
–	Spores more than 3.5 µm long	**4**
3	Stipe present, spines less than 2 mm long	***M. adustulum* (Banker) Ryvarden**
–	Stipe absent, spines up to 4 mm long	***M. sessile***
4	Spines less than 5 mm long, spores less than 5 µm long	**5**
–	Spines up to 10 mm long, spores up to 6.3 µm long	***M. stereoides***
5	Pileal less than 2 cm wide, gloeocystidia present	***M. pusillum* (Brot.) Maas Geest.**
–	Pileal more than 2 cm wide, gloeocystidia absent	**6**
6	Habit on the ground	**7**
–	Habit on the fallen branch of hard wood	**8**
7	Spines more than 3 mm long	***M. africanum* Mossebo & Ryvarden**
–	Spines less than 3 mm long	***M. citrinum* Ryvarden**
8	Pileal margin black, hyphae acyanophilous	***M. adustum***
–	Pileal margin yellowish white, hyphae cyanophilous	***M. subadustum***

## Supplementary Material

XML Treatment for Mycorrhaphium subadustum

XML Treatment for Trullella conifericola
